# Lung cancer in HIV-infected patients

**DOI:** 10.7448/IAS.15.6.18087

**Published:** 2012-11-11

**Authors:** R Palacios, J Lebrón, M Guerrero-León, A Del Arco, J Colmenero, M Márquez, J Santos

**Affiliations:** 1 Hospital Virgen de la Victoria, Málaga, Spain; 2 Hospital Costa del Sol, Marbella, Spain; 3 Hospital Carlos Haya, Málaga, Spain

## Abstract

**Purpose:**

Several studies have shown that HIV patients are at higher risk of lung cancer. Our aim is to analyse the prevalence and features of lung cancer in HIV-infected patients.

**Methods:**

The clinical charts of 4,721 HIV-infected patients seen in three hospitals of southeast Spain (study period 1992–2012) were reviewed, and all patients with a lung cancer were analysed.

**Results:**

There were 61 lung cancers, giving a prevalence of 1.2%. There was a predominance of men (82.0%), and smokers (96.6%; mean pack-years 35.2), with a median age of 48.0 (41.7–52.9) years, and their distribution according to risk group for HIV was: intravenous drug use 58.3%, homosexual 20.0%, and heterosexual 16.7%. Thirty-four (56.7%) patients were Aids cases, and 29 (47.5%) had prior pulmonar events: tuberculosis 16, bacterial pneumonia 9, and *P. jiroveci* pneumonia 4. The median nadir CD4 count was 149/mm^3^ (42–232), the median CD4 count at the time of diagnosis of the lung cancer was 237/mm^3^ (85–397), and 66.1%<350/mm^3^. 66.7% were on ART, and 70% of them had undetectable HIV viral load. The most common histological types of lung cancer were adenocarcinoma and epidermoid, with 24 (40.0%) and 23 (38.3%) cases, respectively. There were 49 (80.3%) cases with advanced stages (III and IV) at diagnosis. The distribution of treatments was: only palliative 23 (39.7%), chemotherapy 14 (24.1%), surgery and chemotherapy 8 (13.8%), radiotherapy 7 (12.1%), surgery 4 (6.9%), and other combined treatments 2 (3.4%). Forty-six (76.7%) patients died, with a median survival time of 3 months. The Kaplan-Meier survival rate at 6 months was 42.7% (at 12 months 28.5%).

**Conclusions:**

The prevalence of lung cancer in this cohort of HIV-patients is high. People affected are mainly men, smokers, with transmission of HIV by intravenous drug use, and around half of them with prior opportunistic pulmonary events. Most patients had low nadir CD4 count, and were immunosuppressed at the time of diagnosis. Adenocarcinoma is the most frequent histological type. The diagnosis is usually made at advanced stages of the neoplasm, and mortality is high.

